# Neural pathways underlying the production of pitch and rhythm in aphasia

**DOI:** 10.1111/nyas.15357

**Published:** 2025-05-11

**Authors:** Anni Pitkäniemi, Teppo Särkämö, Sini‐Tuuli Siponkoski, Noelia Martinez‐Molina, Alicia Lucendo‐Noriega, Nella Moisseinen, Sari Laitinen, Essi‐Reetta Särkämö, Martin Hartmann, Petri Toiviainen, Aleksi J. Sihvonen

**Affiliations:** ^1^ Cognitive Brain Research Unit, Department of Psychology, Faculty of Medicine University of Helsinki Helsinki Finland; ^2^ Centre of Excellence in Music, Mind, Body and Brain University of Jyväskylä & University of Helsinki Jyväskylä & Helsinki Finland; ^3^ Department of Information and Communication Technologies, Centre for Brain and Cognition University Pompeu Fabra Barcelona Spain; ^4^ Department of Music, Art and Culture Studies University of Jyväskylä Jyväskylä Finland; ^5^ Espoo Hospital Espoo Finland; ^6^ Private Choir Conductor Vantaa Finland; ^7^ Queensland Aphasia Research Centre, Surgical, Treatment and Rehabilitation Service The University of Queensland Brisbane Queensland Australia; ^8^ School of Health and Rehabilitation Sciences The University of Queensland Brisbane Queensland Australia; ^9^ Department of Neurology Helsinki University Hospital and Department of Neurosciences, University of Helsinki Helsinki Finland

**Keywords:** aphasia, connectivity, pitch, rhythm, singing

## Abstract

Singing is a universal human attribute. Previous studies suggest that the ability to produce words through singing can be preserved in poststroke aphasia (PSA) and that this is mainly subserved by the spared parts of the left‐lateralized language network. However, it remains unclear to what extent the production of rhythmic–melodic acoustic patterns in singing remains preserved in aphasia and which neural networks and hemisphere(s) are involved in this. In this cross‐sectional study, we set out to investigate the structural neural networks underpinning singing production abilities by combining a whole‐brain white matter correlational tractography approach together with a comprehensive appraisal of pitch, melodic contour, and rhythm production accuracy during both spontaneous and cued singing in a sample of 45 patients with PSA. Our results indicate that PSA patients have poorer singing accuracy (pitch, melodic contour, and rhythm) than matched healthy controls (*N* = 33). The network associated with singing accuracy in aphasia was identified in the left hemisphere–dominant dual stream network involved in auditory‐motor integration of speech, but also extends to multiple other associative and projection pathways, also in the right hemisphere. The results provide insight into alternative communication methods and therapeutic approaches, leveraging music's inherent structure to aid in language recovery and rehabilitation.

## Graphical abstract

Singing is a universal trait, and prior studies suggest singing ability may be preserved in poststroke aphasia. This study examined neural networks underpinning singing in patients with aphasia using white matter connectometry and singing accuracy assessments. Results showed patients with aphasia had poorer singing accuracy than controls, linked to the left‐lateralized dual stream network and other pathways, including the right hemisphere, offering insights for language rehabilitation through music.

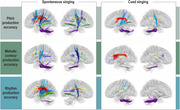

## INTRODUCTION

The relationship between music and language—and their vocal equivalents, singing and speech—has been debated for centuries. While some theories have suggested only a limited or virtually nonexistent connection between the two, even Darwin marked the universality of music in all known cultures, suggesting that a vocal communication system resembling modern singing predated contemporary language.^1^

Singing and speech rely on a shared vocal apparatus and require fine‐grained vocal‐motor control as well as processing of rhythmic–melodic acoustic patterns. Furthermore, songs represent an intriguing interface between language and music as they incorporate linguistic information with melodic and rhythmic elements of music into a consolidated form of expression. As to whether this unified form is also underpinned by integrated processing, the findings have been less consistent.^2^ Nevertheless, the shared characteristics of speech and singing have served as an impetus for developing singing‐based rehabilitation methods, such as melodic intonation therapy, which typically aim to harness the musical features of singing to overcome the deficits associated with speech production.^3^, ^4^, ^5^

Successful singing performance and speech production entails both productive and perceptive processes, which are tightly coupled through several mechanisms, including motor planning and implementation, continuous monitoring of the auditory feedback and associated motor acts, and auditory‐motor mapping,^6^, ^7^, ^8^, ^9^ which have been localized to the bilateral inferior frontal cortex, motor/premotor cortex, and superior temporal cortex.^10^, ^11^, ^12^ Even the theoretical neuroanatomical models of singing^6^, ^7^ propose a neural organization similar to that of speech processing.^13^, ^14^, ^15^, ^16^, ^17^ This dual stream model is composed of a frontoparietal dorsal stream and a frontotemporal ventral stream that are associated with vocal production and auditory perception, respectively, but also engaged with a broader network linked to language and singing processing.^6^, ^7^

Despite the presumed substantial neuroanatomical and behavioral links between singing and speech, recurring case reports from the 1700s onward have attested that patients who have lost their ability to produce spontaneous speech may still be capable to sing the lyrics of a song they know,^18^ suggesting at least partially distinct neural underpinnings for the two domains. Based on melodic intonation therapy studies, it has been proposed that the spared ability to sing in patients with even severe aphasia (and the mechanism through which singing may aid the restoration of speech) is mainly mediated by the contralesional right hemisphere contributions that can be reached and occupied through singing.^3^, ^4^, ^5^, ^19^ However, in contrast to this traditional view, we recently reported that the spared ability to sing the words of a familiar song in aphasia is related to the intactness of cortical temporal regions^20^ and especially the underlying ventral stream structures within the left hemisphere language network as well as more general projection pathways associated with cognitive control and motor functions.^21^ These findings provided direct structural evidence that singing in aphasia is not supported by the contralesional right hemisphere^3^, ^4^, ^5^ but rather by the left‐lateralized language network, which also facilitates speech. These findings also provided the first neuroanatomical explanation as to why deficits in speech and singing words can be distinct or caused by the same focal injury.

These recent advances call for further information on the neural underpinnings of singing production regarding its acoustic features, such as pitch, melody, and rhythm, to paint a more comprehensive picture of singing‐related abilities in aphasia that could be harnessed for rehabilitative purposes. Importantly, in addition to the linguistic component, pitch, melody, and rhythm are fundamental musical components commonly utilized in the existent singing‐based interventions for aphasia. However, at present, there is still controversy surrounding not only the preservation of their production but also the specific neural networks, or even the hemisphere, that underpin them. Perceptive deficits in pitch and rhythm have been linked to right ventral stream disruptions, although less severe and temporary deficits appear to be linked to left‐hemispheric structures.^22^ Interestingly, right ventral stream injuries have been similarly associated with an inability to convey or comprehend affective aspects of speech (i.e., aprosodia), pointing to potential overlaps in the underlying neural networks of music and language processing.^23^

Albeit perception and production are firmly intertwined and their deficits in the realm of music may occur concurrently—typically after a right hemisphere injury^24^, ^25^—cumulating evidence shows that there can also be a mismatch between perception and production, suggesting that their neuroanatomical basis is at least partially divergent. More specifically, difficulties in pitch production can occur in the absence of pitch perception deficits and, inversely, poor‐pitch singing does not necessarily coincide with perception deficits.^8^, ^26^, ^27^ Likewise, case reports suggest that the ability to produce pitch may be distinct from the ability to produce rhythm.^26^, ^28^ More recent neural studies suggest that pitch production in singing primarily involves activity in the bilateral auditory cortices and left frontoparietal regions, while rhythm production relies more on coordination between auditory, motor, and basal ganglia circuits.^29^, ^30^ Moreover, modern models point to extensive networks rather than focal regions underpinning singing performance.^6^, ^7^ Therefore, determining the white matter correlates of different subtask measures is a viable option for elaborating the networks supporting the subcomponents of singing production, but this has been scarcely explored.

Therefore, in the present study, we aimed to (1) investigate whether the accuracy in pitch, melodic contour, and rhythm in singing production is preserved in patients with aphasia when compared to matched controls and (2) determine the white matter pathways associated with both spontaneous and cued production accuracy of pitch, melodic contour, and rhythm in singing among patients with aphasia. To do this, we recorded the spontaneous and cued singing production of patients with poststroke aphasia (PSA) and their healthy, demographically matched controls and analyzed their singing performances with an acoustic analysis tool providing pitch, melodic contour, and rhythm accuracy scores. Subsequently, the acoustic analysis scores of the patients with aphasia were combined with whole‐brain multishell diffusion MRI correlational tractography that allows the determination of the white matter pathways associated with accuracy in each subcomponent (pitch, melodic contour, and rhythm) and type of spontaneous and cued production. We hypothesized that (1) patients with PSA have spared ability to produce pitch, melodic contour, and rhythm of a familiar song through both spontaneous and cued production, determined by equal error rates with matched healthy controls; (2) the neural pathways underlying pitch, melodic contour, and rhythm production are different, at least partially, from the language network; and (3) the structural network involved in pitch production is different from that of underlying rhythm production.

## MATERIALS AND METHODS

### Subjects

A total of 45 patients with PSA (25 females, mean age = 64.4, SD = 10.2) and 33 demographically matched neurologically intact controls (20 females, mean age = 62.1, SD = 9.9) were recruited. The demographic and clinical information of these patients with PSA is presented in Table 1. In either group, subjects had no professional background in music.

**TABLE 1:** Demographic and clinical characteristics of the patients with PSA (*N* = 45) and healthy controls (*N* = 33).

	Patients with PSA	Healthy controls	*p*‐value
**Demographics**			
Age (years)	64.4 (10.2)	62.1 (9.9)	0.250 (t)
Sex (female/male)	25/20	15/18	0.378 (χ^2^)
Handedness (right/left/both)	39/5/1	33/0/0	0.092 (χ^2^)
Education (years)	14.3 (4.1)	15.9 (4.3)	0.060 (U)
**Music background**			
Music lessons (years)	0.4 (1.4)	0.3 (1.2)	0.653 (U)
Choir singing (years)	3.1 (8.9)	3.6 (4.8)	0.073 (U)
MBEA (correct %)	70.9 (13.1)		
**Clinical background**			
Time from stroke (years)	9.0 (7.7)		
Lesion volume (cm^3^)	95.8 (87.6)		
BDAE severity rating	3.0 (1.4)		
WAB Aphasia Quotient	63.4 (34.1)		
**Speech and spoken language production outcomes**			
Connected spoken language production^a^ efficacy^b^	30.8 (25.5)		
Spoken repetition efficacy^b^	46.0 (27.1)		
BDAE verbal agility	7.2 (4.6)		
**Singing outcomes**			
Connected singing efficacy^b^	42.0 (28.8)		
Sung repetition efficacy^b^	23.9 (14.5)		

*Note*: Data are reported in mean (SD) unless otherwise specified.

^a^ Correct information units per minute.

^b^ Correct words per minute.

Abbreviations: BDAE, Boston Diagnostic Aphasia Examination; MBEA, Montreal Battery of Evaluation of Amusia; PSA, poststroke aphasia; WAB, Western Aphasia Battery.

Subjects with aphasia were recruited from the Helsinki and Turku regions during 2017–2019 through patient organizations (Helsinki‐Uusimaa and Turku region Stroke Association, Finnish Brain Association) and clinical speech therapists. All subjects with PSA had chronic aphasia (>6 months since onset) due to left hemisphere stroke, as confirmed by clinical assessment. They were all over 18 years of age, spoke Finnish as a native language, and had no hearing deficits. They also had no severe cognitive impairments that could affect their ability to cooperate and provide informed consent, no history of substance abuse, and no neurological or psychiatric comorbidities.

Neurologically intact controls were selected from a dataset of a cross‐sectional study on singing and healthy aging.^31^ Control subjects were recruited from across Finland during 2019–2020 through advertisements (public venues, email lists, and social media channels) as well as through direct contact with amateur‐level choirs. From an original dataset of *N* = 100 healthy subjects, a subsample of 33 control subjects were matched with patients by age, sex, education years, and previous singing experience at group level. Control subjects were over 20 years of age and right‐handed, spoke Finnish as a native language, and had no diagnosis of a hearing impairment, language or neurological disorder, cognitive decline, or dementia. For control subjects, behavioral tasks were carried out by a graduate student (in psychology) trained for the task by a psychologist.

The study was conducted in accordance with the Declaration of Helsinki and approved by the Ethics Committees of the Hospital Districts of Helsinki‐Uusimaa and Southwest Finland, European Research Council Executive Agency (ERCEA), and the University of Helsinki Ethical Review Board in the Humanities and Social and Behavioural Sciences. An informed consent was obtained from all recruited subjects.

### Behavioral assessment

For patients with PSA, behavioral tasks were completed as a part of a baseline neuropsychological assessment (taking approximately 2 h) of a randomized controlled clinical trial and were administered by a trained psychologist. Clinical information about the severity of the aphasia and language deficits were assessed with the Boston Diagnostic Aphasia Examination (BDAE)^32^ severity rating and the Western Aphasia Battery (WAB)^33^ Aphasia Quotient (AQ). The aphasia severity of each subject was initially evaluated by the recruiting psychologist and confirmed over the behavioral assessment.

Connected spoken language production efficacy was calculated based on a composite score of mean correct information units per minute produced during three different spontaneous speech production tasks: (1) WAB picture description task (“Picnic”),^33^ (2) sequential picture description (“Argument”),^34^ and (3) personal information (responding to the question “Tell me what you usually do on Sundays”).^34^ The participants were encouraged to continue spoken language production for at least one minute in each task. There was no upper limit for the production time. The participants were not provided with hints to prompt production. Correct information units with minor phonemic paraphasia were admitted, provided that the content was still clearly recognizable.

The spoken and sung repetition task was assessed with a task of 16 prerecorded phrases that were presented to the subjects through headphones. First, the subjects listened to and repeated the phrases presented with normal prosody (spoken format). In the second round, identical phrases were presented and repeated with melodic intoning (sung format). The length of the phrases varied from 1 word (e.g., “Huomenta,” transl. “Good morning”) to 5 words (e.g., “Ylös, kengät paina ei jalkaa,” transl. “Get up, shoes don't weigh you down”), resulting in a total of 42 words. The length of spoken stimuli varied from 1.07 to 3.95 s and of sung stimuli from 2.02 to 7.90 s, in line with the fact that production rates are typically faster for speech than singing.^35^ The first repetition attempt of each phrase was used for scoring. To yield a production rate for repetition tasks, the number of correct words (with minor phonemic paraphasia accepted) per minute was calculated. There were no missing data. The BDAE articulatory agility task was also conducted to attain an estimate of motor speech difficulties.

In the singing task administered to both patients with PSA and healthy matched controls, the subjects were first asked to sing a familiar song (*Jaakko‐kulta*, the Finnish version of *Frere Jacques/Brother John*) from memory and from any key the individual subject was comfortable singing the song, two times successively. After this, the subjects were asked to sing the same song again two times consecutively, but now in unison with an auditory model that was presented to them through headphones. Separate auditory models for female and male subjects were provided to ensure a more optimal scale for singing. The singing efficacy score was determined by the number of correctly sung words produced per minute.

Singing production was digitally recorded with a lightweight earset microphone (DPA d:fine Dual Earset Omni Tan). Recordings were acquired, and the cued auditory model was presented with Presentation software (Neurobehavioral Systems, Inc., www.neurobs.com). The digital recordings of the singing performance of two subjects with aphasia were lost because of a technical malfunction.

The audio recordings were minimally processed by converting them from stereo to mono signals while keeping the original sample rate (48 kHz). Then, all recordings were listened to by authors N.M.M. and A.L.N., and internal coughing, laughter, long pauses, syllable repetitions, exhalations, and interjections that could interfere with the performance of the geometric matching algorithm (see the next section) were removed from the recordings. In order to determine the exact syllables produced by each participant (total syllables = 64 including both repetitions) and create a subject‐specific musical instrument digital interface (MIDI) template of the song as a benchmark for the acoustic analysis, the syllables of each recording were manually annotated using Praat software (https://www.fon.hum.uva.nl/praat/) in combination with the ProsodyPro algorithm (https://www.homepages.ucl.ac.uk/~uclyyix/ProsodyPro/). The ProsodyPro algorithm was used to obtain the names (i.e., syllables) for each labeled interval as manually annotated in Praat for each recording in a separate file. This file allowed the automated creation of a subject‐specific template with a custom MATLAB script. Briefly, we used the resulting *.label file from ProsodyPro to determine the indices of the syllables that were potentially missing from each participant and singing production and created a subject‐specific MIDI template of the song “Brother John” where the MIDI notes corresponding to the missing syllables were excluded. When all syllables were produced, the original MIDI template was used.

### Acoustic analysis

We have developed a fully automated technique using MATLAB (Natick, Massachusetts) for estimating pitch and rhythmic accuracy in audio, which is based on preexisting knowledge about a song. This knowledge is represented as a geometric description of the song's melody in the time–pitch plane. By representing a melody as a geometric shape, we can measure melodic dissimilarity in terms of the geometric difference in these two dimensions between two melodies. Our approach offers two key advantages. First, the task of segmenting the melody into musical notes can be automated using a geometric pitch template of the song. Second, geometric matching algorithms can provide meaningful measures of pitch error (for example, expressed in semitones). This is particularly useful when compared to string matching algorithms, which quantify dissimilarity based on the minimum‐weight series of operations required to transform one melody into another.^36^

**FIGURE 1:**
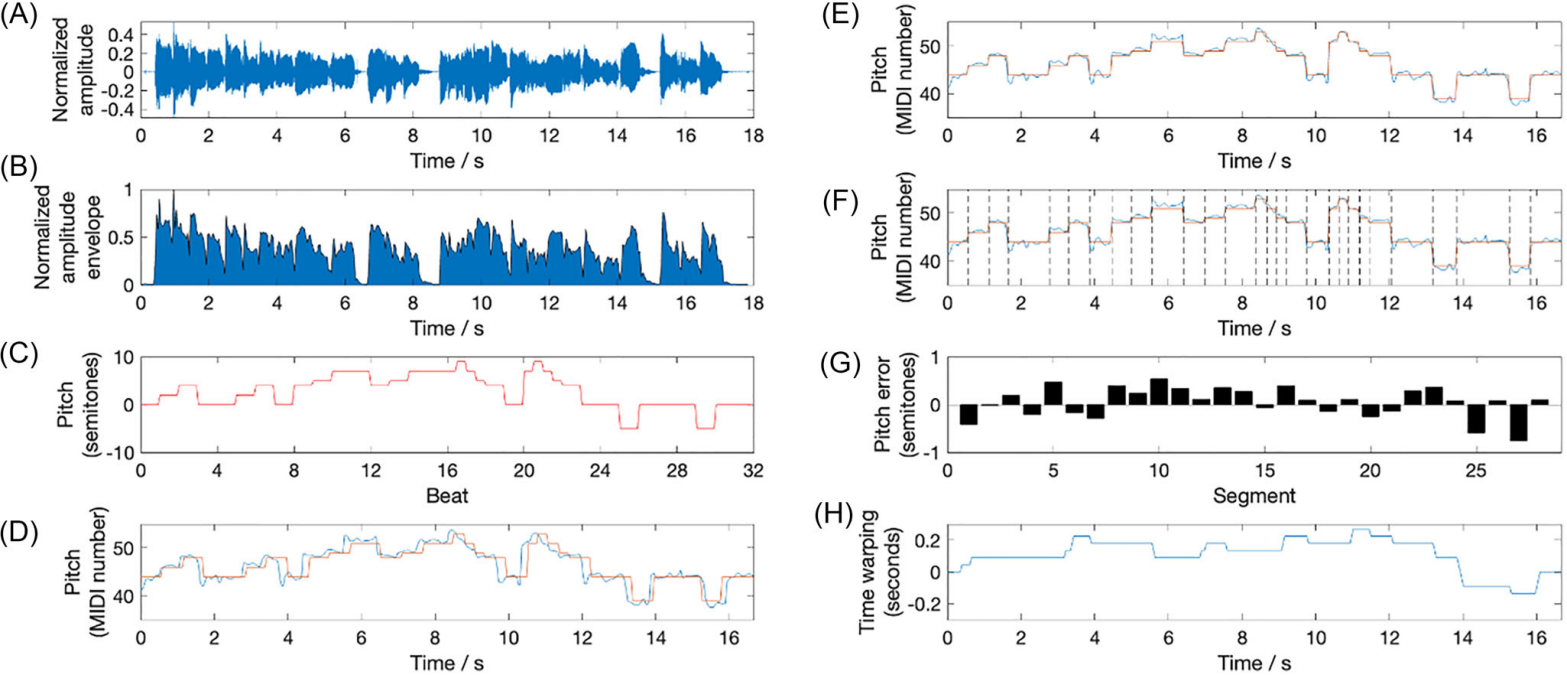
Estimating pitch and rhythmic accuracy of a singing recording. (A) Audio waveform of the recording; (B) estimated audio envelope, used to trim the silent portions at the beginning and end; (C) geometric pitch template of the song “Brother John” used as a benchmark; (D) estimated pitch contour of the recording (blue), superimposed with the time‐stretched and pitch‐shifted template (red); (E) estimated pitch contour (blue) and the pitch template (red) dynamically time‐warped to optimally match each other; (F) same as previous, with segment boundaries (dashed lines) placed at the discontinuities of the pitch template; (G) estimated pitch error for each segment, derived by the difference between the median estimated pitch and the template pitch; and (H) warping path obtained from the dynamic time warping algorithm and used to determine the rhythmic error score.

The process of obtaining a pitch, melody, and rhythm error score for each audio recording (Figure 1A) begins with the estimation of its pitch contour in MIDI note numbers (Figure 1C). This is achieved using the YIN algorithm (threshold for trimming = 0.1; minf_0_ = 30, maxf_0_ = 1000, hop size = 32).^37^ Subsequently, silent sections at the beginning and end of the pitch curve are removed. This is accomplished by extracting a normalized audio envelope (Figure 1B) and selecting a central portion of the pitch curve. This selected portion has a median amplitude envelope magnitude that exceeds 10% of the maximal waveform amplitude. The subsequent stages of the analysis depicted in Figure 1 involve using the geometric pitch template of the song “Brother John” as a benchmark. The melody notes are encoded in semitones relative to the tonic and in the smallest time unit of the song (eighth notes). For example, the first bar of the song (“Are you sleep‐ing?”) is represented as 0 0 2 2 4 4 0 0. Next, the template is time‐stretched using previous neighbor interpolation to match the pitch curve length, and vertically translated by shifting its mean pitch to that of the pitch curve (Figure 1D).

As singing is never rhythmically absolutely accurate, when comparing the pitch curve with the geometric pitch template, it is necessary to temporally align these two signals. This alignment is accomplished by stretching them using Euclidean dynamic time warping^38^ (Figure 1E).

The pitch error measure quantifies the average distance in semitones between two time–pitch “piano‐roll” representations. To obtain this measure, pitch outliers are first identified and removed by selecting portions of the pitch curve and the template where the absolute signal difference is within 2.5 standard deviations. The template is then pitch‐shifted once more to match the pitch curve. Inter‐onset segments are subsequently derived based on pitch changes in the template (Figure 1F). Finally, the pitch error in semitones is calculated as the mean absolute difference between the median pitch of each segment in the pitch curve and the corresponding pitch value in the geometric pitch template (Figure 1E).

The melody contour error measure provides a coarser description of pitch accuracy. It compares the pitch curve and the template by examining the differences in pitch change direction between successive segments. This pitch change direction encoding is called Parsons code.^39^ Initially, the pitch direction between segments is coded for the template and the quantized pitch curve, which is obtained via median averaging (as referenced in the pitch error calculation above). Subsequently, the L¹ distance, or the sum of absolute differences, between these two pitch direction curves is obtained. This measure provides an understanding of the overall accuracy of the pitch in terms of its directional changes.

The rhythmic inaccuracy of the singing, in relation to the metronomic template, is calculated following the dynamic time warping step. This method involves estimating the degree of dynamic time warping, which includes temporal stretching and compression, needed to align the pitch curve with the metronomic template. The values at each time point, as shown in Figure 1H, represent the amount of temporal shift applied to the estimated pitch contour to achieve optimal alignment with the pitch template. The rhythmic error score is then determined as the average absolute rate of change observed in the warping path. This score provides a measure of the rhythmic accuracy of the singing, expressed in units of the time unit used in the template (eighth notes).

### MRI data acquisition

Subjects with PSA were scanned on a 3T Siemens Skyra scanner at the Helsinki and Uusimaa Hospital District Medical Imaging Center or at the Medical Imaging Centre of Southwest Finland. Multishell diffusion‐weighted MRI (dMRI) with 13 non‐diffusion–weighted volumes and 130 diffusion weighted volumes (30 volumes with *b* = 1000 s/mm^2^ and 100 volumes with *b* = 2500 s/mm^2^) were obtained with the following parameters: repetition time (TR) = 5000 ms, echo time = 104 ms, field of view = 240 × 240 mm, voxel size = 2.0 × 2.0 × 2.0 mm^3^. All participants were prescreened for contraindications for MRI upon recruitment and again immediately before scanning. There was no missing data. A sample of 45 participants should provide a power of at least 0.8 to detect relationships between language skills and white matter connectivity patterns as suggested by previously reported data on white matter connectivity in PSA.^40^

### MRI data preprocessing and reconstruction

The dMRI data preprocessing and reconstruction were conducted as described in detail previously.^21^ First, the data was denoised using the Marchenko‐Pastur PCA method^41^ implemented in the MRTrix3 software.^42^ Then, Gibbs ringing artefact correction was performed.^43^ After this, the dMRI data were reconstructed into the Montreal Neurological Institute space in DSI Studio (http://dsi‐studio.labsolver.org) using q‐space diffeomorphic reconstruction^44^ with a 1.25 diffusion sampling length ratio, permitting the construction of spin distribution functions (SDFs)^45^ in spatially normalized diffusion data. The b‐table orientation was monitored with an automatic quality control routine using the fiber coherence index.^46^ The *R* ^2^ values indicating the goodness‐of‐fit between template and anisotropy map were inspected for each participant. The spatial output resolution of SDF maps was 2.0 × 2.0 × 2.0 mm^3^. To verify normalization quality, forceps major and minor were used as an anatomical benchmark and manually explored,^40^ and the resulting anisotropy maps were inspected individually in Montreal Neurological Institute space and reported previously.^21^ Restricted diffusion imaging was used to quantify restricted diffusion.^47^ Derived from the peak orientations on SDF, quantitative anisotropy (QA) was extracted as the local connectome fingerprint^48^ to be used in the connectometry analysis.

### Correlational tractography and statistical analysis

The dMRI correlational tractography utilizing connectometry^49^ analyses were performed with DSI Studio. Correlational tractography, through the use of connectometry, statistically maps and analyzes the degree of connectivity between adjacent voxels within a white matter pathway (i.e., local connectomes) defined by the density of the diffusing spins (SDFs). Connectometry tracks the segments of a white matter fascicle that exhibits significant association with the study variable, as compared to mapping the association between a study variable and mean fractional anisotropy in a voxel or representing a whole tract. As the dMRI data are reconstructed into standard space and tracking is based on template, it also minimizes bias induced by manual tracking. Six multiple regression models were performed to explore local connectomes associated with (1) pitch, (2) melodic contour, and (3) rhythm errors during spontaneous singing (*N* = 36; Pitch_Spont_, Melody_Spont_, and Rhythm_Spont_, respectively), and (4) pitch, (2) melodic contour, and (3) rhythm errors during singing along model (*N* = 44; Pitch_Cued_, Melody_Cued_, and Rhythm_Cued_, respectively). QA‐values were normalized to homogenize the data to account for two scanning sites. Given the six calculated regression models, the false discovery rate (FDR) threshold was set to 0.0083 (Bonferroni correction). Due to the set Bonferroni correction (*p* < 0.0083) and the lowest degrees of freedom (*n* = 35) in the analysis of connected singing in the low spoken language production efficacy group, conversion of *p*‐value to *t*‐value (one‐tailed) equals 2.516. Therefore, QA‐aided deterministic fiber tracking algorithm^50^ at a T‐score threshold of 2.5 was used to track trajectories and obtain correlational tractography. Topology‐informed pruning^51^ with four iterations was applied to filter false fiber trajectories. Tracks with a length threshold exceeding 20 voxel distance were selected. Two thousand randomized permutations were run to obtain the null distribution of the track length and subsequently estimate the FDR. The pattern of results (i.e., significant tracts or tract segments) were inspected, tracts constituting of single spurious fibers likely to be identified due to statistical noise were removed, and robust tract segments were separated. Separate robust tract segments were first identified by an automated method implemented in DSI Studio and then checked visually by two of the authors (A.P. and A.J.S.) separately. When necessary, the Human Connectome Project template HCP 1065‐based tracking was consulted to make sure the correct fiber bundle for classification. Furthermore, for reporting purposes, the tracts were grouped based on the nomenclature presented in the Atlas of Human Brain Connections,^52^ but referred to as dorsal stream when belonging to the perisylvian network (arcuate fasciculus, superior longitudinal fasciculus) and ventral stream when belonging to the inferior network (inferior longitudinal fasciculus, inferior fronto‐occipital fasciculus and uncinate).

## RESULTS

### Performance of the subjects

**FIGURE 2:**
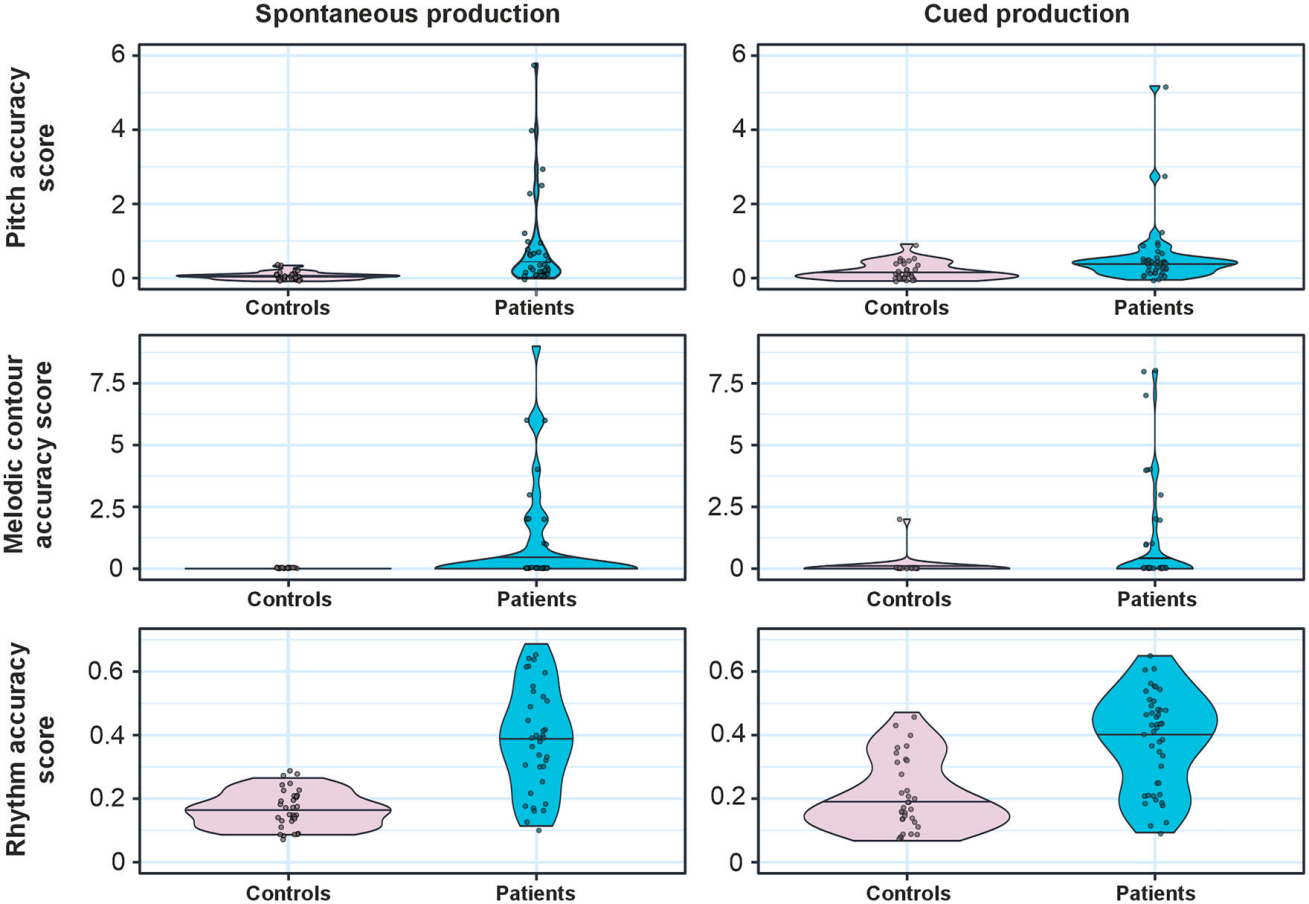
Violin plots showing the distribution of the data for spontaneous and cued pitch, melodic contour, and rhythm accuracy scores for healthy controls and patients. The straight black line refers to the group median. Higher scores denote more errors.

First, using Mann–Whitney *U* tests, we evaluated whether PSA affects the patients’ pitch, melody, and rhythm accuracy during singing. In all the evaluated singing measures (pitch, melody contour, and rhythm), patients with PSA were less accurate than the healthy matched controls in both spontaneous singing (Pitch_Spont_: *U* = 1021.00, *P*_FDR_ < 0.001; Melody_Spont_: *U* = 759.00, *P*_FDR_ = 0.001; Rhythm_Spont_: *U* = 1065.00, *P*_FDR_ < 0.001) and cued singing (Pitch_Cued_: *U* = 1070.00, *P*_FDR_ < 0.001; Melody_Cued_: *U* = 904.00, *P*_FDR_ = 0.005; Rhythm_Cued_: *U* = 1184.00, *P*_FDR_ < 0.001). For mean singing accuracy scores, see Figure 2 and Table 2. Within patients with PSA, the error scores for pitch, melody contour, and rhythm all correlated significantly (Table 3).

**TABLE 2:** Singing accuracy scores of the patients with aphasia and healthy controls.

	Patients (*N* = 45)	Controls (*N* = 33)
Pitch_Spont_	0.98 (1.20)	0.27 (0.10)
Melody_Spont_	1.00 (2.11)	0.00 (0.00)
Rhythm_Spont_	0.38 (0.16)	0.16 (0.05)
Pitch_Cued_	0.88 (1.23)	0.39 (0.22)
Melody_Cued_	1.02 (2.15)	0.06 (0.29)
Rhythm_Cued_	0.38 (0.15)	0.19 (0.10)

*Note*: Data are reported as mean (SD). Higher scores denote more errors.

**TABLE 3:** Associations of singing accuracy measures in patients with aphasia.

	Pitch_Spont_	Melody_Spont_	Rhythm_Spont_	Pitch_Cued_	Melody_Cued_	Rhythm_Cued_
Pitch_Spont_	‐	**0.750 (0.001)**	**0.770 (0.001)**	**0.824 (0.001)**	**0.429 (0.009)**	**0.490 (0.002)**
Melody_Spont_	**0.750 (0.001)**	‐	**0.619 (0.001)**	**0.608 (0.001)**	**0.368 (0.027)**	**0.405 (0.014)**
Rhythm_Spont_	**0.770 (0.001)**	**0.619 (0.001)**	‐	**0.756 (0.001)**	**0.367 (0.028)**	**0.412 (0.013)**
Pitch_Cued_	**0.824 (0.001)**	**0.608 (0.001)**	**0.756 (0.001)**	‐	**0.545 (0.001)**	**0.646 (0.001)**
Melody_Cued_	**0.429 (0.009)**	**0.368 (0.027)**	**0.367 (0.028)**	**0.545 (0.001)**	‐	**0.468 (0.001)**
Rhythm_Cued_	**0.490 (0.002)**	**0.405 (0.014)**	**0.412 (0.013)**	**0.646 (0.001)**	**0.468 (0.001)**	‐

*Note*: All results are Spearman's rho and reported as *r*_s_ (*p*). Bold values are statistically significant (*p* < 0.05).

### Clinical, behavioral, and demographical associations of singing accuracy measures

To evaluate the relationship between the singing accuracy measures and the clinical, demographic, and behavioral information of the subjects, Spearman (two‐tailed) correlations were calculated (*n* = 78). Due to the exploratory nature of these correlations, *p*‐values were not corrected for multiple comparisons. Statistical values for all correlations are presented in Table 4.

**TABLE 4:** Clinical, behavioral, and demographical associations of singing accuracy measures.

	Spontaneous			Cued		
	Pitch	Melody	Rhythm	Pitch	Melody	Rhythm
Age	−0.265 (0.119)	−0.192 (0.261)	−0.119 (0.492)	**−0.314 (0.038)**	**−0.343 (0.023)**	−0.076 (0.626)
Education	**−0.473 (0.004)**	−0.300 (0.076)	**−0.369 (0.027)**	**−0.370 (0.014)**	−0.027 (0.863)	−0.291 (0.055)
						
MBEA score	−0.308 (0.068)	−0.272 (0.108)	**−0.360 (0.031)**	−0.108 (0.486)	−0.071 (0.647)	0.026 (0.865)
Choir singing (years)	**−0.554 (0.001)**	−0.292 (0.083)	**−0.352 (0.035)**	**−0.415 (0.005)**	−0.242 (0.113)	−0.258 (0.091)
						
Time from stroke	**−0.394 (0.023)**	−0.257 (0.149)	**−0.468 (0.006)**	−0.095 (0.555)	−0.187 (0.243)	−0.053 (0.743)
Lesion volume	0.148 (0.390)	−0.025 (0.887)	0.123 (0.474)	0.057 (0.716)	**0.300 (0.048)**	0.056 (0.719)
BDAE severity rating	0.049 (0.776)	0.246 (0.148)	−0.026 (0.882)	0.023 (0.884)	−0.284 (0.062)	−0.018 (0.908)
WAB Aphasia Quotient	−0.296 (0.205)	0.339 (0.144)	−0.352 (0.128)	−0.075 (0.727)	−0.140 (0.515)	−0.134 (0.534)
BDAE verbal agility	−0.160 (0.350)	0.003 (0.985)	−0.136 (0.428)	−0.028 (0.855)	−0.219 (0.153)	−0.225 (0.143)
						
Connected spoken language production efficacy	**−0.338 (0.044)**	−0.159 (0.353)	**−0.424 (0.010)**	−0.175 (0.256)	**−0.333 (0.027)**	−0.266 (0.080)
Singing efficacy	0.033 (0.852)	0.046 (0.794)	0.017 (0.921)	−0.040 (0.798)	−0.199 (0.202)	−0.296 (0.054)
Spoken repetition efficacy	−0.099 (0.566)	0.069 (0.691)	−0.204 (0.233)	−0.040 (0.796)	−0.184 (0.232)	−0.170 (0.271)
Sung repetition efficacy	0.138 (0.423)	0.182 (0.287)	0.021 (0.902)	0.177 (0.250)	−0.122 (0.430)	0.012 (0.941)

*Note*: All results are Spearman's rho and reported as *r*_s_ (*p*). Bold values are statistically significant (*p* < 0.05).

Abbreviations: BDAE, Boston Diagnostic Aphasia Examination; MBEA, Montreal Battery of Evaluation of Amusia; WAB, Western Aphasia Battery.

Older age correlated with fewer errors in Pitch_Cued_ (*r*_s_ = −0.314, *p* = 0.038) and Melody_Cued_ (*r*_s_ = −0.343, *p* = 0.02). Greater education years correlated with fewer errors in Pitch_Spont_ (*r*_s_ = −0.473, *p* = 0.004), Rhythm_Spont_ (*r*_s_ = −0.369, *p* = 0.027), Pitch_Cued_ (*r*_s_ = −0.370, *p* = 0.014), but not with other measures.

Higher Montreal Battery of Evaluation of Amusia (MBEA) scores correlated only with fewer errors in Rhythm_Spont_ (*r*_s_ = −0.360, *p* = 0.031), whereas greater choir singing experience in years correlated with fewer errors in Pitch_Spont_ (*r*_s_ = −0.554, *p* < 0.001), Rhythm_Spont_ (*r*_s_ = −0.352, *p* = 0.035), and Pitch_Cued_ (*r*_s_ = −0.415, *p* = 0.005), but not with other singing measures.

Lower lesion volume showed only a weak correlation with fewer errors in Melody_Cued_ (*r*_s_ = 0.300, *p* = 0.048), but not with any other singing measure whereas longer time from stroke showed weak‐to‐moderate correlations with fewer errors in Pitch_Spont_ (*r*_s_ = −0.394, *p* = 0.023) and Rhythm_Spont_ (*r*_s_ = −0.468, *p* = 0.006), but not with other singing measures.

Clinical measures of speech outcome (BDAE severity, WAB AQ, or BDAE verbal agility score) were not significantly associated with any of the singing measures (*p* = 0.062−0.985). Better connected spoken language production efficacy, as measured by the correct words per minute, correlated with fewer errors in Pitch_Spont_ (*r*_s_ = −0.338, *p* = 0.044), Rhythm_Spont_ (*r*_s_ = −0.424, *p* = 0.010), and Melody_Cued_ (*r*_s_ = −0.333, *p* = 0.027), but not with other singing measures. Spoken repetition efficacy did not show significant correlations with any of the singing accuracy measures (*p* = 0.232−0.796).

Singing efficacy or singing repetition efficacy did not correlate significantly with any of the singing measures (*p* = 0.056−0.941).

In summary, the clinical, behavioral, and demographic information of the subjects did not correlate consistently with any of the singing accuracy measures. Yet, better connected spoken language production efficacy was associated with fewer errors in three singing measures, similar to longer education and greater choir singing experience. Rhythm_Spont_ was associated with most background variables (five), whereas Melody_Spont_ and Rhythm_Cued_ were not associated with any of the background variables.

### Networks supporting pitch production accuracy

**FIGURE 3:**
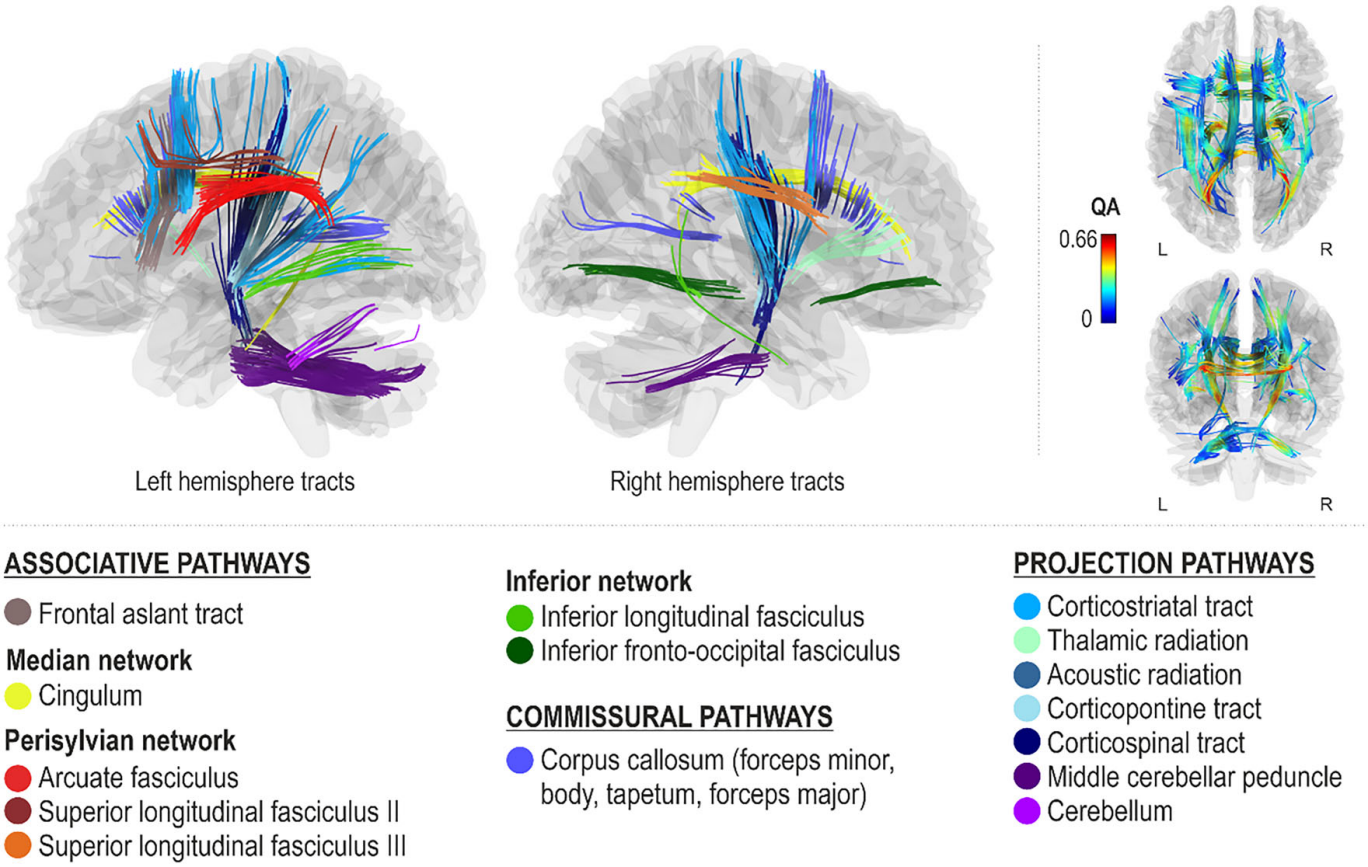
Networks supporting spontaneous pitch production accuracy.

**FIGURE 4:**
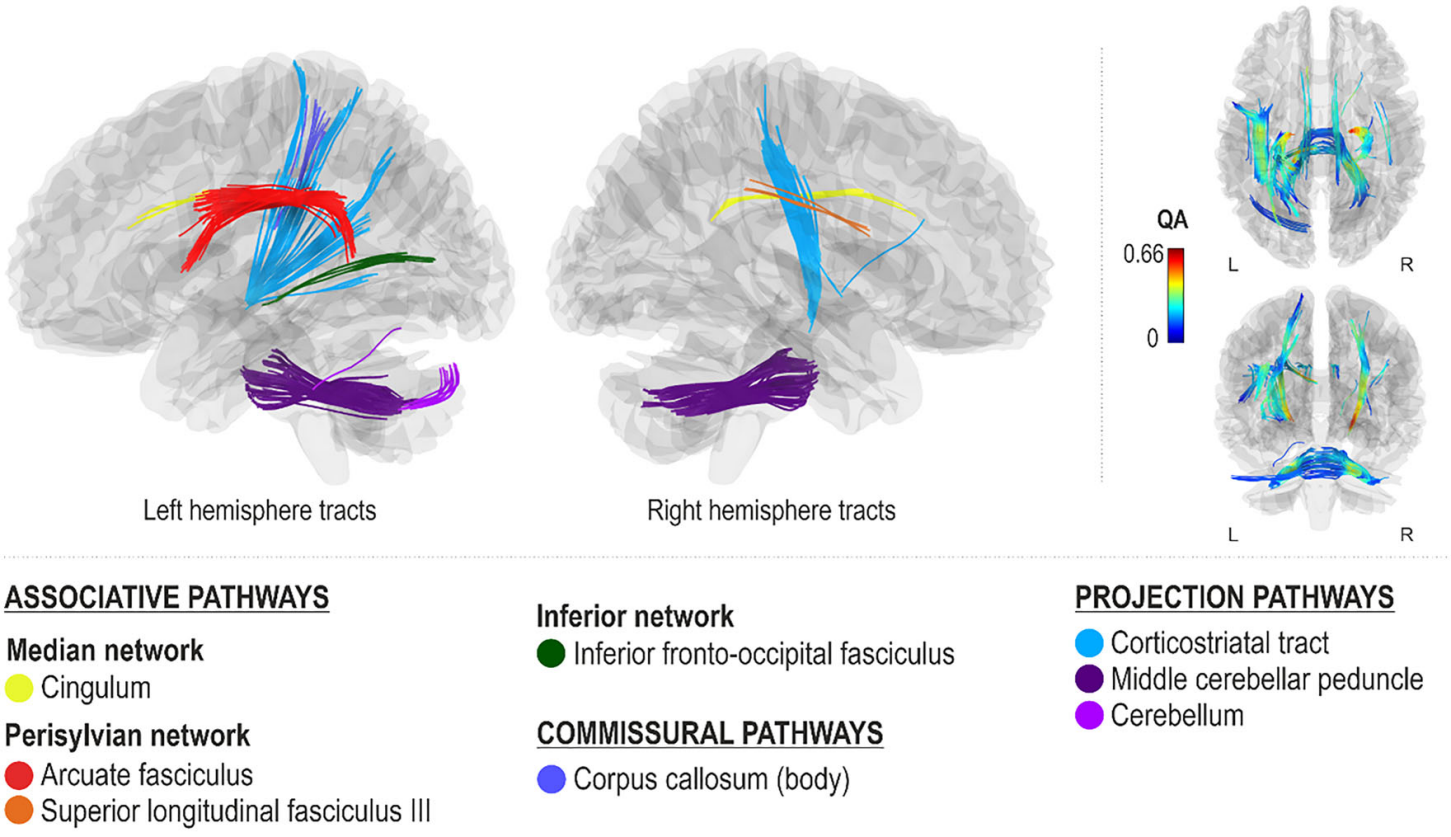
Networks supporting cued pitch production accuracy.

Better spontaneous (Pitch_Spont_) and cued (Pitch_Cued_) pitch production accuracy, as measured by fewer errors in singing individual pitches, were both associated with greater QA in the dorsal stream (left arcuate fasciculus and right superior longitudinal fasciculus III), left ventral stream (left inferior fronto‐occipital fasciculus), median network (cingulum bilaterally), projection pathways (bilateral corticostriatal tracts and middle cerebellar peduncles and left cerebellum), and commissural pathways (corpus callosum body) (FDR < 0.0083; Figures 3 and 4). Additionally, better spontaneous pitch production accuracy was associated with greater QA in the left dorsal stream (left superior longitudinal fasciculus II), right ventral stream (right inferior fronto‐occipital fasciculus and inferior longitudinal fasciculus), associative pathways (left frontal aslant tract), projection pathways (bilateral corticospinal tracts, left corticopontine tract, and right thalamic radiation), and commissural pathways (corpus callosum forceps minor, tapetum, and forceps major) (FDR < 0.0083; Figure 3).

### Networks supporting melodic contour production accuracy

**FIGURE 5:**
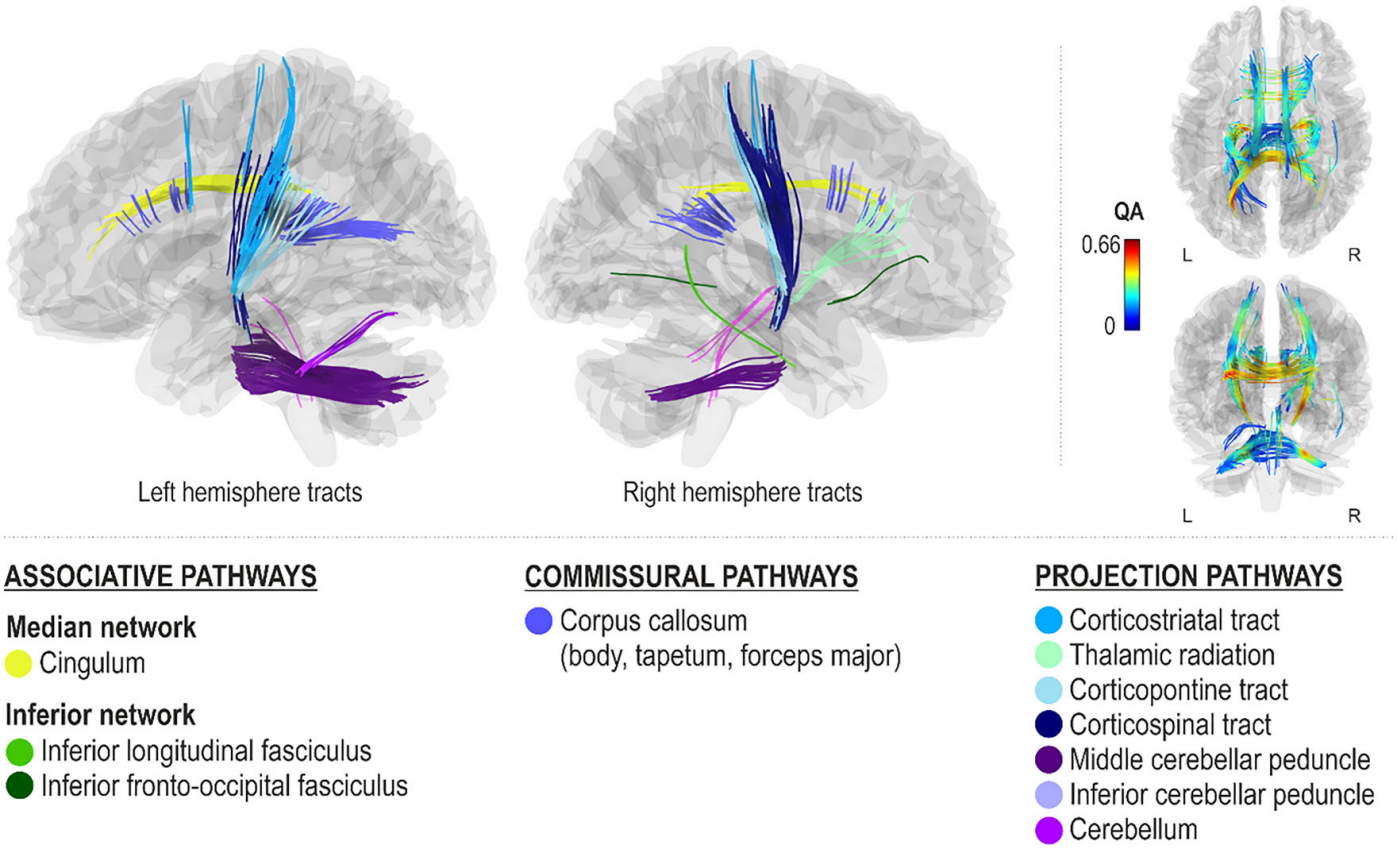
Networks supporting spontaneous melodic contour production accuracy.

**FIGURE 6:**
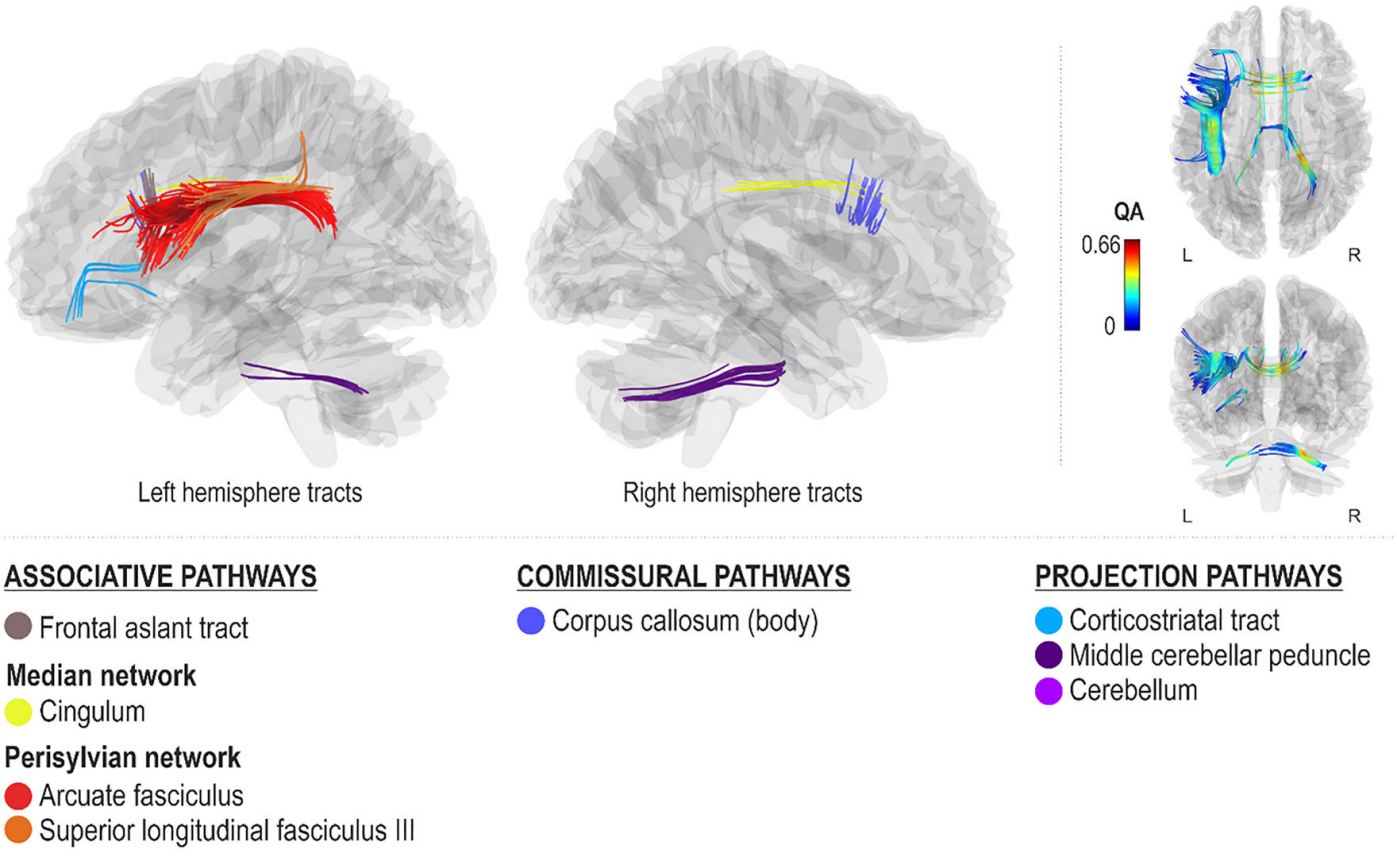
Networks supporting cued melodic contour production accuracy.

Better spontaneous (Melody_Spont_) and cued (Melody_Cued_) melodic contour production accuracy, as measured by fewer errors in singing correct consecutive pitch directions, were both associated with greater QA in the median network (cingulum bilaterally), projection pathways (left corticostriatal tract and bilateral middle cerebellar tracts), and commissural pathways (corpus callosum body) (FDR < 0.0083; Figures 5 and 6). Additionally, better spontaneous melodic contour production accuracy was associated with greater QA in the right ventral stream (right inferior longitudinal fasciculus and inferior fronto‐occipital fasciculus), projection pathways (right corticostriatal tract, bilateral corticospinal and tracts, bilateral superior cerebellar peduncles, left inferior cerebellar peduncle and cerebellum, and right thalamic radiation), and commissural pathways (corpus callosum forceps major) (FDR < 0.0083; Figure 5). Better cued melodic contour production accuracy, in turn, was additionally associated with greater QA in the left dorsal stream (left arcuate fasciculus and superior longitudinal fasciculus III) and associative pathways (left frontal aslant tract) (FDR < 0.0083; Figure 6).

### Networks supporting rhythm production accuracy

**FIGURE 7:**
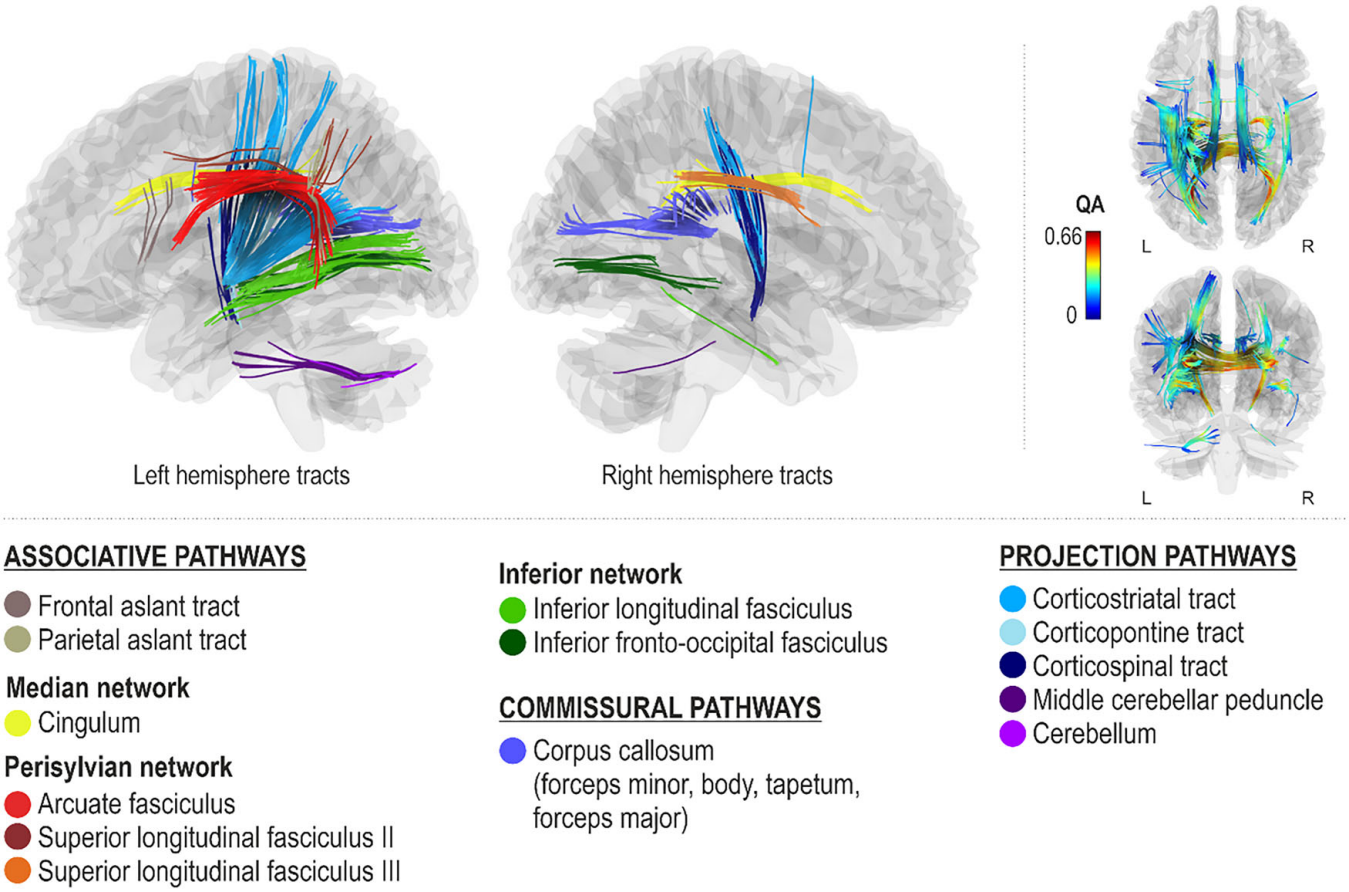
Networks supporting spontaneous rhythm production accuracy.

**FIGURE 8:**
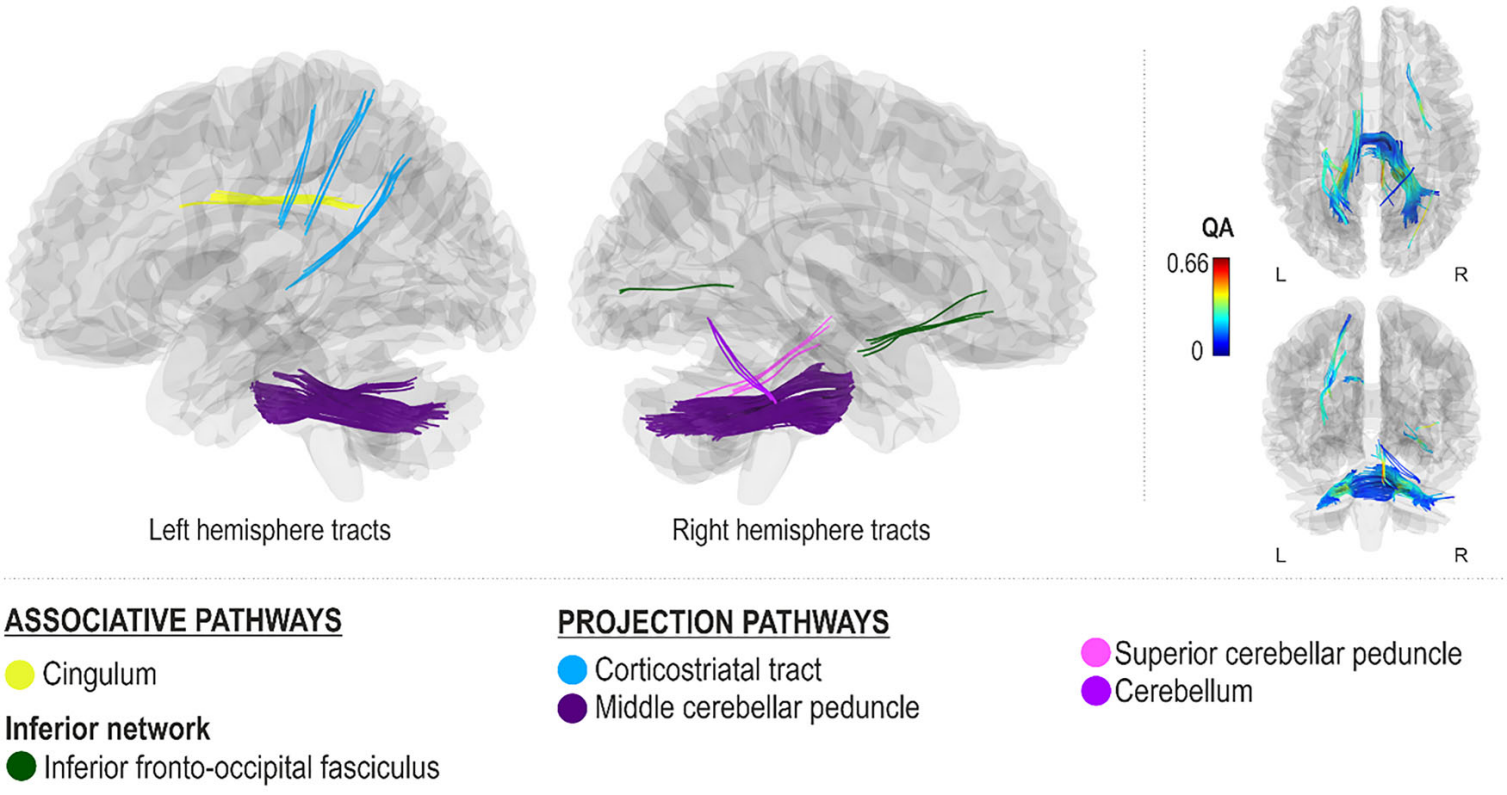
Networks supporting cued rhythm production accuracy.

Better spontaneous (Rhythm_Spont_) and cued (Rhythm_Cued_) rhythm production accuracy, as measured by lower onset time differences with respect to a metronomic template during spontaneous singing, were both associated with greater QA in the right ventral stream (right inferior fronto‐occipital fasciculus), left median network (left cingulum), and projection pathways (left corticostriatal tract and bilateral middle cerebellar peduncles) (FDR < 0.0083; Figures 7 and 8). Additionally, better spontaneous rhythm production accuracy was associated with greater QA in the dorsal stream (left arcuate fasciculus and superior longitudinal fasciculus II and right superior longitudinal fasciculus III), ventral stream (left inferior fronto‐occipital fasciculus and bilateral inferior longitudinal fasciculi), left associative tracts (left frontal and parietal aslant tracts), right median network (right cingulum), projection pathways (right corticostriatal tract, bilateral corticospinal tracts, and left corticopontine tract and cerebellum), and commissural pathways (corpus callosum body, tapetum and forceps major) (FDR < 0.0083; Figure 7). Better cued rhythm production accuracy, in turn, was additionally associated with greater QA in the projection pathways (right superior cerebellar peduncle and cerebellum) (FDR < 0.0083; Figure 8).

## DISCUSSION

The primary objectives of this study were to explore whether the accuracy in pitch, melodic contour, and rhythm production in singing is preserved in patients with aphasia when compared to matched controls and to determine the neural circuits subserving pitch, melodic contour, and rhythm production during both spontaneous and cued singing. Together with multishell dMRI data and sensitive QA‐based whole‐brain white matter connectometry analyses, we had patients with aphasia as well as matched healthy controls sing an over‐learned song to minimize dependence on reading lyrics and on learning ability, and to constitute a universally replicable outcome measure. Our main findings indicate that (1) patients with aphasia have poorer singing accuracy than matched healthy controls across multiple acoustic features, (2) the singing accuracy is mainly associated with connected spoken language production efficacy, and (3) the neural circuitry associated with singing accuracy is composed of the left hemisphere–dominant dual stream network involved in auditory‐motor integration of speech, but also multiple other associative and projection pathways, also in the right hemisphere.

Our behavioral analyses failed to detect the unperturbed ability to produce the pitch, melodic contour, and rhythm of a familiar song through both spontaneous and cued production in aphasia. In fact, in all measured singing variables (pitch, melodic contour, rhythm), both spontaneous and cued, people with aphasia performed significantly worse than the matched healthy controls. This aligns with previous observations that patients with left hemisphere stroke exhibit impaired speech auditory feedback error processing during speech pitch‐shift alterations.^53^ These deficits in speech error detection in aphasia are also associated with repetition deficits, but not with deficits in fluency, auditory verbal comprehension, or naming.^54^ In the present study, clinical measures of aphasia severity, verbal agility, and spoken and sung repetition efficacy were not associated with any of the singing accuracy measures. Interestingly, the production rate of song lyrics (connected singing efficacy) was not correlated with simultaneous pitch, melodic, or rhythmic production accuracy. This finding implies that the production of lyrics and musical features of singing can be preserved on dissociable levels in aphasia, at least in the instance of singing an over‐learned song, in which the production of words is based on more automatized processes, perhaps resembling nonpropositional or formulaic speech. However, and in contrast, connected spoken language production efficacy, as measured by the correct information units per minute, was significantly associated with spontaneous singing pitch and rhythm accuracy as well as cued melody contour performance. This finding links the pitch and rhythm production abilities with the core speech processing deficit in nonfluent aphasia, and further refines the previous proposal^54^ that the mechanisms for performing tasks involving sensorimotor integration may be shared between not only speech and language systems but also with singing, another avenue for vocalization.

The present findings align with previous endeavors on the neural structures supporting singing but provide novel structural evidence on the extent of involved neural circuits. Functional neuroimaging studies in healthy subjects have provided evidence that singing production recruits a widespread network of brain regions, including the anterior cingulate cortex, bilateral auditory, prefrontal, somatomotor, and somatosensory cortices, basal ganglia, and cerebellum.^29^, ^55^, ^56^, ^57^ The network's activity has not been reported to be dependent on the complexity of the singing task. Most interestingly, the anterior cingulate cortex and left dorsal and ventral stream structures activate more when subjects aim to correct singing a single tone with pitch‐shifted auditory feedback.^29^ The left‐lateralized activity shift during correcting singing output under pitch‐shifted auditory feedback might reflect the left dorsal stream structures’ involvement in selecting responses due to sensorimotor associations, while the right dorsal stream structures are more recruited when selecting an alternative to or reprogramming an already‐learned response.^58^ However, our study did not utilize pitch‐shifted auditory feedback during singing. Yet, the left dorsal stream structures were more implicated in the present results than their right counterparts. This could be explained by the automatic triggering of corrective motor adjustments during spontaneous and cued singing, with left dorsal stream structures involved in vocal adjustments to maintain correct pitch output.

In accordance with the dual‐stream model of speech,^14^, ^59^ the auditory feedback error processes are managed through primarily left‐dominant dorsal stream that encompass sensorimotor regions located in the left frontal, parietal, and temporal cortices, crucial for auditory‐motor integration of speech. In turn, damage to this network, especially to the inferior frontal, precentral, inferior parietal, and superior temporal cortices, leads to impaired vocal auditory feedback processing and control.^60^ The explorations on the specific structural neural circuitry affected in speech error detection during vocal production in general have been, however, scarce in aphasia. The present results revealed a widespread structural network in association with pitch, melodic contour, and rhythm production accuracy. More specifically, the left‐dominant dorsal stream, especially the arcuate fasciculus, which has been previously associated with impaired auditory feedback processing in aphasia, was implicated in impaired spontaneous and cued pitch production accuracy (Pitch_Spont_ and Pitch_Cued_), spontaneous rhythm production accuracy (Rhythm_Spont_), and cued melodic contour production accuracy (Melody_Cued_). Furthermore, the left ventral stream network, especially the inferior fronto‐occipital fasciculus, was implicated in impaired spontaneous and cued pitch production accuracy (Pitch_Spont_ and Pitch_Cued_) as well as in spontaneous rhythm production accuracy (Rhythm_Spont_). These findings suggest that similar computational neural resources, the left dorsal and ventral stream networks, support vocal feedback control, regardless of the nature of the vocalization.^6^ Within this network, as discussed by Behroozmand et al., the left dorsal stream detects errors to drive compensatory vocal behavior, and the left ventral stream may serve as a mechanism for processing errors to update neural representations of the internal forward model in the inferior frontal cortex.^54^ Similar structural circuits are also implicated in both connected spoken language production and connected singing production (i.e., efficacy of singing correct words),^21^ further underlining how the left hemisphere dorsal and ventral stream networks support vocal production in all forms. Interestingly, these stream networks were less associated with cued than spontaneous production accuracy, especially for rhythm, which suggests that singing along an auditory model reduces requirements for vocal‐motor representations, perhaps explaining, at least partially, why patients with PSA can benefit from choral singing.^61^

Beyond the left dual stream system, dorsal and ventral stream networks in the right hemisphere also seem to support singing accuracy. Right dorsal stream was implicated in impaired spontaneous and cued pitch production accuracy (Pitch_Spont_ and Pitch_Cued_) and in spontaneous rhythm production accuracy (Rhythm_Spont_) whereas the right ventral stream network was implicated in impaired spontaneous and cued rhythm production accuracy (Rhythm_Spont_ and Rhythm_Cued_) as well as in spontaneous melodic contour production accuracy (Melody_Spont_), which interestingly did not show any association to the left ventral stream or to the dorsal stream in either hemisphere. Previously, the right ventral stream and associated regions have been implicated in perception^19^ and production^10^ of melodies. Studies on auditory feedback control of pitch have shown greater involvement of right dorsal and ventral stream regions than left hemispheric areas.^62^, ^63^ These findings suggest that both the left and the right hemispheres contribute to vocal production control, but feedforward control is predominantly subserved by the left hemisphere dual stream system, whereas auditory feedback control is subserved more by the right hemisphere dual stream network.^64^ Yet, it is possible that for unperturbed performance, both hemispheres are needed to operate in concert to allow for efficient feedforward and feedback control. A previous functional MRI study assessing the production of affective and nonaffective vocalizations revealed a highly overlapping set of brain regions, suggesting that these areas are nodes in a single vocal network, with an emphasis on pitch modulation.^65^ During singing, pitch modulation and control are especially important, and interestingly, both left and right dorsal streams—which interconnect bilaterally to the vocal network nodes^65^—were implicated in both Pitch_Spont_ and Rhythm_Spont_. These findings parallel with previous evidence indicate that speech motor control involves feedback error detection in auditory cortices that subsequently activate motor‐related areas for the adjustment of speech parameters during speaking.^9^

For the effective utilization of both hemispheres during singing production and related vocal control, the information needs to be transferred interhemispherically via callosal fibers. In accordance with this, the corpus callosum was implicated in almost all singing accuracy measures in the present study, albeit less in cued than spontaneous production, perhaps owing to the reduced need for hemispheric crosstalk when following an auditory model. A previous study has shown that reduced structural connectivity between the hemispheres is associated with poststroke amusia, a music perception deficit, especially in the spectral (i.e., pitch) domain.^66^ Furthermore, the strength of callosal auditory pathways has been associated with better performance in an auditory speech perception task.^67^ The hemispheres might still contribute differently to vocal production. For example, the perception of acoustic‐rhythmic information in music and speech (i.e., prosody) rely both on the right hemisphere dual stream system, especially the right ventral stream.^23^, ^66^, ^68^, ^69^ However, further processing of acoustic‐rhythmic information in speech requires a crosstalk between the hemispheres to combine the prosodic and syntactic information in language.^70^ Taken together, this evidence suggests that both the left and right dual stream systems are involved in singing accuracy. It seems that singing accurately requires the processing and feedback control of acoustic‐rhythmic information in the right hemisphere, which is then transferred to the left hemisphere for syntactic integration of lyrical information via callosal fibers, and finally combining the abstract representations and sensorimotor information to be integrated with these representations.^16^ However, as the present results and previous evidence suggest, the critical—but not the only—network for vocal production, including singing, appears to reside in the left hemisphere. This may be due to the integration of linguistic and acoustic‐rhythmic information, as well as the need for fine‐grained vocal‐motor control, which depends on the left sensorimotor speech areas. This would explain the observed behavioral results together with the most consistent structural findings, including the association of pitch and rhythm production accuracy with the left aslant tract, one of the core pathways linked to motor speech production.^71^

One of the novel findings in this study was the consistent involvement of bilateral cingulum in all singing accuracy measures; that is, greater QA was consistently associated with fewer errors in pitch, melodic contour, and rhythm, both in spontaneous and cued production. Previous studies have shown increased anterior cingulate cortex activity during affective vocalization, correlating with vocal acoustics during expressive speech.^72^, ^73^ However, while implicated in affective vocalization, more specific functional neuroimaging endeavors have revealed that the cingulate cortex is activated regardless of whether the vocalizations were affective,^65^ including singing.^74^ Cingulum might also be implicated in the present results due to its strong association with learning, memory, and emotion processing.^75^ Due to the singing assessment used, patients were required to sing a well‐learned song from memory spontaneously, which necessitates mental imagery of the song and retrieval and utilization of its musical and linguistic information from memory. However, cingulum was also implicated in all cued singing measurements that do not require similar memory processing, but more matching of singing output with the presented singing representation. Therefore, the abovementioned presented results and evidence suggest that cingulum is part of the vocal‐motor system^72^, ^73^ involved in singing accuracy.

In addition to the abovementioned white matter tracts and circuits, multiple other tracts were associated with singing accuracy in the present study. This most likely reflects the complexity of singing production versus simple phonation used in many previous studies on auditory error detection. One of the pronounced neural circuits involved in singing accuracy, especially the rhythm accuracy, was the motor network, including striatocortical projection pathways and cerebellum. Both basal ganglia and the cerebellum are involved in music processing, especially in the processing of rhythm.^76^, ^77^ A recent study demonstrated that internalized rhythms are maintained as periodic neuronal activity within the cerebellum and striatum, preferring sensory and motor representations, respectively.^77^ Cerebellar degeneration has also been shown to inhibit the propensity to covertly adapt to other people's speech rate and rhythm.^78^ Furthermore, the cerebellum and basal ganglia contribute to efficient decoding of and response to vocal emotions.^79^ According to animal studies, the cerebellum also influences vocal timing, and the neural circuit interconnecting the cerebellum and basal ganglia contributes to vocal learning.^80^ For these reasons, the cerebellar tracts were most significantly associated with cued rhythm production accuracy, where matching one's vocal performance to a model is required.

Overall, while certain aspects of singing may show lateralization to one hemisphere or the other, singing is a complex behavior that likely involves the coordinated activity of both hemispheres of the brain. But is one more critical than the other? Yes, our previous evidence suggests that the left hemisphere is critical to singing words.^20^, ^21^ However, based on the present findings, the picture is less clear regarding the lateralization of rhythmic‐acoustic production accuracy during singing. The present findings link a widespread structural network with singing accuracy, including both left and right dual stream systems and callosal fibers. Still, aphasia was associated with poorer singing accuracy compared to healthy matched controls, but the production of lyrics and musical features of singing were dissociated. This reflects the fact that lesions that give rise to aphasia are located within the network involved in controlling singing accuracy, as they induce perturbations to singing performance. But are the lesions overlapping with the critical hubs, brain regions crucial for enabling neuronal signaling and communication,^81^ for singing accuracy? Previous case studies have reported that right temporal stroke severely impairs pitch reproduction abilities in professional musicians.^24^, ^82^ Moreover, while the network involved in music perception is widespread, comprising both hemispheres,^83^, ^84^, ^85^ yet focal spatially distributed lesions disrupting music processing map to a common brain network that centers on the right superior temporal cortex.^86^ Taken together, this evidence suggests that the critical hubs for singing accuracy could also be located in the right hemisphere. Yet, lesions giving rise to aphasia are located within this network, albeit it is centered on the right hemisphere, hypothetically inducing functional and structural perturbations and the observed impaired singing accuracy. This explanation would also help us to understand the widespread structural findings and the disparate behavioral correlations of singing accuracy in the present study. Future studies utilizing multimodal neuroimaging data comparing the left and right hemisphere–damaged patients are necessary to provide more fine‐grained evidence regarding singing accuracy and to tease apart its crucial substrates.

There are several limitations in the present study to be considered when interpreting the results. First, the singing accuracy assessment was based on participants singing a well‐known song. This was done to achieve a reliable measure of preserved singing skills in individuals with aphasia, as this approach circumvents the need for reading lyrics, which is often impaired in aphasia, and minimizes the influence of working memory. This allowed us to concentrate on the act of singing production, which was the primary focus of our study. However, whether the neural architectures involved in accurate singing, both linguistically and musically, of novel songs in aphasia completely overlap or extend beyond the observed neural patterns to include learning‐related remains to be explored by future research. Second, the present study utilized acoustic analyses of pitch, melodic contour, and rhythm accuracies during singing with lyrics. Whether the same neural structures support singing without lyrics, utilizing, for example, a singular syllable or humming, remains to be determined. Third, while the present study pinpoints the neural circuits involved in accurate singing, the present singing assessment prevents us from dissecting the contributions of different white matter pathways on the auditory feedback processes during singing. This would require an experimental design similar to studies evaluating vocal production under pitch‐shifted altered auditory feedback mapping the auditory neural responses.^54^ However, based on the present findings and previous evidence, a similar network most likely subserves auditory feedback mapping during all vocal production. Finally, the present study did not assess singing accuracy in aphasic patients separately across different levels of aphasia severity and lesion size or across time; larger studies with more extensive samples, including also more severe cases, and follow‐up assessments are warranted. From the clinical perspective (and given the observed correlation between singing accuracy and connected spoken language production efficacy), the potential predictive value of damage/preservation of the singing production network for the efficacy of singing‐based rehabilitation in aphasia still needs to be explored.

## CONCLUSION

Our results suggest that the left hemisphere–dominant dual stream system is involved in singing accuracy, as evidenced by individuals with aphasia demonstrating lower singing accuracy compared to matched healthy controls and singing accuracy being primarily linked to the connected spoken language production efficacy. Furthermore, the neural circuitry underlying singing accuracy is widespread, including both left and right dual stream systems and callosal fibers necessitating the processing and feedback control of acoustic‐rhythmic information, further syntactic integration of lyrical information, and finally combining the abstract representations and sensorimotor information to be integrated with these representations. Furthermore, cingulum and various associative and projection pathways, including motor‐related circuits, are implicated in singing accuracy. These findings link singing accuracy with other vocal production abilities behaviorally and neurally, which, an evolutionary perspective, support theories of the parallel development of speech and singing, complying with a conservation principle that the mammalian brain optimally develops using minimum expenditure of wiring costs and optimal conductivity speed.^87^

## AUTHOR CONTRIBUTIONS

Anni Pitkäniemi, Teppo Särkämö, Sini‐Tuuli Siponkoski, Noelia Martinez‐Molina, Alicia Lucendo‐Noriega, Nella Moisseinen, Sari Laitinen, Essi‐Reetta Särkämö, Martin Hartmann, Petri Toiviainen, and Aleksi J. Sihvonen designed this study and wrote the paper. Anni Pitkäniemi and Sini‐Tuuli Siponkoski performed assessments. Anni Pitkäniemi, Teppo Särkämö, Noelia Martinez‐Molina, Alicia Lucendo Noriega, and Aleksi J. Sihvonen analyzed the data.

## CONFLICT OF INTEREST STATEMENT

The authors declare no conflicts of interest.

## PEER REVIEW

The peer review history for this article is available at https://publons.com/publon/10.1111/nyas.15357.

## Data Availability

The data that support the findings of this study are available on request from the corresponding author. The data are not publicly available due to privacy or ethical restrictions. All software and packages, their versions, and relevant specifications are stated in the Methods section.
